# Ozone-Induced Responses in *Croton floribundus* Spreng. (Euphorbiaceae): Metabolic Cross-Talk between Volatile Organic Compounds and Calcium Oxalate Crystal Formation

**DOI:** 10.1371/journal.pone.0105072

**Published:** 2014-08-28

**Authors:** Poliana Cardoso-Gustavson, Vanessa Palermo Bolsoni, Debora Pinheiro de Oliveira, Maria Tereza Gromboni Guaratini, Marcos Pereira Marinho Aidar, Mauro Alexandre Marabesi, Edenise Segala Alves, Silvia Ribeiro de Souza

**Affiliations:** 1 Programa de Pós-Graduação em Biodiversidade Vegetal e Meio Ambiente, Instituto de Botânica, São Paulo, São Paulo, Brazil; 2 Núcleo de Pesquisa em Ecologia, Instituto de Botânica, São Paulo, São Paulo, Brazil; 3 Núcleo de Pesquisa em Fisiologia e Bioquímica, Instituto de Botânica, São Paulo, São Paulo, Brazil; 4 Núcleo de Pesquisa em Anatomia, Instituto de Botânica, São Paulo, São Paulo, Brazil; Centro de Investigación y de Estudios Avanzados, Mexico

## Abstract

Here, we proposed that volatile organic compounds (VOC), specifically methyl salicylate (MeSA), mediate the formation of calcium oxalate crystals (COC) in the defence against ozone (O_3_) oxidative damage. We performed experiments using *Croton floribundus,* a pioneer tree species that is tolerant to O_3_ and widely distributed in the Brazilian forest. This species constitutively produces COC. We exposed plants to a controlled fumigation experiment and assessed biochemical, physiological, and morphological parameters. O_3_ induced a significant increase in the concentrations of constitutive oxygenated compounds, MeSA and terpenoids as well as in COC number. Our analysis supported the hypothesis that ozone-induced VOC (mainly MeSA) regulate ROS formation in a way that promotes the opening of calcium channels and the subsequent formation of COC in a fast and stable manner to stop the consequences of the reactive oxygen species in the tissue, indeed immobilising the excess calcium (caused by acute exposition to O_3_) that can be dangerous to the plant. To test this hypothesis, we performed an independent experiment spraying MeSA over *C. floribundus* plants and observed an increase in the number of COC, indicating that this compound has a potential to directly induce their formation. Thus, the tolerance of *C. floribundus* to O_3_ oxidative stress could be a consequence of a higher capacity for the production of VOC and COC rather than the modulation of antioxidant balance. We also present some insights into constitutive morphological features that may be related to the tolerance that this species exhibits to O_3_.

## Introduction

Tropospheric ozone (O_3_) is considered to be the gaseous pollutant that is most damaging to plants due to its strong oxidation capacity [1]. This gas initially enters plants through the stomata, causing biochemical and physiological alterations [2]; based on the concentration, the duration of the exposure, and the responsiveness of the plant species, O_3_ can induce disturbances in organelles and tissues, leading to visible morphological symptoms [3]–[9].

A biochemical balance is required in the cell to counteract the negative effects of O_3_. Enzymatic and non-enzymatic antioxidant compounds, including ascorbate peroxidase (APX), superoxide dismutase (SOD), peroxidase (POD), and ascorbic acid (AA), counteract the increase in reactive oxygen species (ROS) promoted by O_3_ [2], [10]–[11]. Another possible metabolic response against oxidative stress that is actively modulated by plants is the production of terpenoids, which can constitute the emissions of volatile organic compounds (VOC) [12], [13]. Being antioxidant compounds, VOC can remove the ROS formed in the intercellular spaces [11]–[12], [14]–[22]. Mono- and sesquiterpenes are the most dominant VOC emitted by plants in response to O_3_ [1], [23]–[25]; they constitute a large family of plant metabolites, with diverse functions in plant growth, development and stress response [26]. These compounds can be produced by various metabolic routes [27], preferentially in phloem and xylem parenchyma or by secretory cells associated with these tissues [28]–[30].

Intracellular calcium is also modulated during plant defence responses to O_3_ [31], which might lead to the formation of calcium salt crystals. Calcium oxalate crystals (COC) have roles in the defence against herbivores and/or the accumulation of excess calcium –see reviews in [32]–[34]. It is well known that these constitutive inclusions can be quantitatively induced by biotic stressors such as herbivory [35]–[37]. Although there is only one report of the induction of crystal formation by O_3_ in a gymnosperm species, *Picea abies* [38], there seems to be a significant difference in the quantity of COC produced in *Eugenia uniflora* (Myrtaceae), a tropical species, when polluted and non-polluted environments are considered [39].

There are few studies on the responses of tropical Brazilian tree species to O_3_ stress [40]. *Croton floribundus* Spreng. is a pioneer tree species widely distributed in the Brazilian forest and recommended for use in ecological restoration [41]. This species seems to be tolerant to O_3_ fumigation, not presenting any structural symptoms of oxidative stress even under 80 ppb of O_3_ 6 h/day during 53 days [42], although visible symptoms such as hypersensitive-like responses (HR-like), peroxide hydrogen accumulation (H_2_O_2_) and polyphenol compound accumulation occurred after exposure to 200 ppb of O_3_ for 3 h/day for three days [8]. In our ongoing research, O_3_ has not been able to change the antioxidant levels in this species, reinforcing the hypothesis that *C. floribundus* is tolerant to oxidative stress caused by this pollutant (at least under less-intense acute exposures). Moreover, in our ongoing research, VOC and COC levels appeared to be O_3_-dependent. These preliminary findings suggested that the responsiveness of *C. floribundus* to O_3_ might be linked with VOC emission and COC formation. Thus, we raised the hypothesis of which there is a metabolic cross-talk between VOC emission and COC formation that confers defences against oxidative stress.

To test this hypothesis, the aims of this study are (1) to assess ozone oxidative stress by measuring biochemical and physiological responses; (2) to verify whether the variation in the emitted VOC and in the quantity of COC co-occur as a response to this pollutant and if so, (3) to evaluate whether there is cross-talk between the emitted VOC and the COC formation, by the direct application of a compound present in the emitted *bouquet* that is probably involved in the induction of the COC formation.

## Materials and Methods

### Plant material

Six-month old *C. floribundus* plants were purchased from BIOvida Company (São Paulo, Brazil) and immediately planted in 10 L pots filled with a 3∶1 mixture of peat and sand, and watered by capillarity. Plants were kept inside the greenhouse for three weeks and then were transferred from the greenhouse to the fumigation chambers, where they were kept for 2 days before the beginning of fumigation experiment (acclimation period).

### Using ozone fumigation to assess leaf responses

The ozone fumigation experiments were performed in closed chambers kept inside a laboratory with temperature and humidity controlled with central air conditioner and under artificial illumination supplied by metallic vapour (400 W) and fluorescent (30 W TL05) lamps [43]. The material was divided into two lots, with half exposed to ozone (FA+O3) and the other half receiving filtered air (FA) only. The chambers were composed of a stainless steel structure covered by a film of Teflon, in the dimensions of 85 cm×94 cm×85 cm (W×D×H). The air was filtered by paper filter to remove gross particulate matter (Whatman 40), followed by silica gel (150 g, Merck), active carbon (250 g, Merck), potassium permanganate (500 g, Purafil Select), and paper filter to remove fine particulate (Whatman QMA). The filtration efficiency was assessed by measuring ozone levels in the air passed through the filtering system. The average ozone levels reached a maximum of 5 ppb after filtration, which indicates an efficiency of filtration of 98.5% [44]. After filtration, the air was enriched with 80 ppb of ozone. Ozone was generated under electrical discharge by the dissociation of oxygen contained in filtered air, using an ozone generator (Ozontechenic). The ozone levels were monitored using an Ecotech 9810B photometric monitor. The ozone monitor was calibrated once before each exposure. During the fumigation experiments, the mean temperature, relative humidity, and photon flux density values were 29±1.5°C, 63±17 and 184.2 µmol/m^2^.s respectively, simulating appropriate conditions for optimal growth of *C. floribundus*. In this experiment, six plants were exposed to filtered ozone-free air (FA) and another six to filtered air plus 80 ppb of ozone (FA+O3) for 4 hours/day per seven consecutive days. Each experiment was performed in triplicate. In order to reduce chamber effects, plants were switched between two chambers in the end of every day of exposure. Thus, both chambers were used for FA and FA+O3 treatment. The position of the plants was also changed to counteract the positional effect [45]. It is important to highlight that there were no differences in the conditions (illumination, humidity, temperature) in which the experiments were carried out.

### Biochemical and physiological responses to ozone: fluorescence and antioxidant analyses

A Pulse-Amplitude-Modulated Fluorometer (PAM-2100, Heinz Walz GMBH, Effeltrich, Germany) was used to measure leaf steady-state chlorophyll fluorescence parameters after seven days of fumigation and following a 30 min adaptation to the dark. The NPQ was calculated as NPQ = (Fm–Fm′)/Fm′, where Fm is a maximal fluorescence in the dark and Fm′ is a maximal fluorescence in the light. The Fv/Fm values (photosynthetic quantum efficiency) represent averages from 15 measurements taken sequentially on two different fully expand leaves of three plants per experiment. Electron Transport Rate (ETR) is calculated as ETR = yield × 0.5 × 0.82 × PAR, where yield is the quantum yield of the PSII (Fq/Fm), 0.5 represents the light absorbed by the PSII, 0.82 represents the absorbance of the leaf and PAR represents the light intensity used (400 µmol photons/m^2^.s^2^ using the halogen light source). Values of ETR represent ten measurements in two different fully expand leaves of three plants per treatment. All measurements were taken in the morning, between 10∶00 and 11∶00 am.

The antioxidant defences were analysed in six individuals per treatment. Collection of leaves and preparation of extracts for analysis of antioxidants always followed the same sequence in time to avoid diurnal variation. The extraction was carried out with a mix of all expanded leaves. Total ascorbic acid was measured in 0.5 g of fresh leaves and homogenised with 12 mL of EDTA-Na_2_ (0.07%) and oxalic acid (0.5%). The mixture was centrifuged at 40.000 g for 30 min at 2°C. An aliquot of the supernatant was added to 2.5 mL of DCPIP (0.02%), and absorbance was measured with a spectrophotometer at 520 nm. After the addition of 0.05 mL of ascorbic acid (1%), a second absorbance measurement was taken. Both absorbance measurements were used to estimate the total ascorbic acid content following [46].

Superoxide dismutase activity was measured in 0.35 g of fresh leaves homogenised with 12 mL of potassium phosphate buffer (50 mM pH 7.5), EDTA-Na_2_ 1 mM, NaCl 50 mM and ascorbic acid 1 mM in the presence of 0.4 g of PVPP 2%. This mixture was centrifuged at 22.000 g for 25 min at 4°C. SOD activity was assayed by measuring the SOD inhibition of the NBT photochemical reduction [47]. Each reaction mixture contained 0.5 mL of EDTA-Na_2_ 0.54 mM, 0.8 mL of potassium phosphate buffer (0.1 M, pH 7.0), 0.5 mL of methionine 0.13 mM, 0.5 mL of NBT 0.44 mM, 0.2 mL of riboflavin 1 mM, and 0.2 mL of leaf extract. The samples were incubated for 20 min under a fluorescent lamp (80 W). The absorbance of the reaction mixture was measured at 560 nm. A similar mixture lacking the leaf extract was used as a control, and a dark control mixture served as a blank. The enzymatic activity was expressed as the amount of extract needed to inhibit the reduction of NBT by 50%.

Peroxidase activity was measured in 0.3 g of leaves homogenised with 12 mL potassium phosphate buffer (0.1 M, pH 7.0) in the presence of 0.4 g of PVPP 2%. The homogenate was centrifuged at 40.000 g for 30 min at 2°C. Peroxidase activity was also measured in a reaction mixture of plant extracts using 0.1 M potassium phosphate buffer (pH 5.5) and phenylenediamine (1%) to which an aliquot of H_2_O_2_ (0.3%) was added. Unspecific POD activity was measured with a spectrophotometer following the increase in absorbance (DA) at 485 nm due to the formation of an H_2_O_2_-POD complex at two different times in the linear reaction curve [48].

### Volatile Collection

Volatiles from the headspace of six whole plants from each treatment (FA and FA+O3) were collected into steel tubes containing 150 mg charcoal adsorbent (Supelco, PA, USA) at an airflow rate of approximately 200 ml/min for 60 min. To avoid the VOC from soil, the vessels were covered with aluminium foil. During the collection pure air was continuously supplied into the chambers. One tube was fixed into each chamber (FA and FA+O3) with a line Teflon tubes and connected in the vacuum pumps for volatiles sampling. All collections were performed in the morning after approximately 60 min of the light being switched on and before starting the ozone fumigation. The ozone scrubbers (filters coated with a saturated solution of potassium iodide) were fixed before the adsorbent to avoid any degradation of samples by residual ozone. The collections were made every day after the first day of exposure, comprising a total of six samples per treatment (n = 6). Samples were replicated twice. Blank samples from an empty chamber were also collected twice.

### CG-MS analyses

VOC were analysed by gas chromatography-mass spectrometry (GC-MS Hewlett-Packard GC 6890, MSD 5973, Wilmington, DE, USA). Trapped compounds were desorbed chemically with 200 µL of hexane-methylchloride (4∶1). The volume was reduced to 50 µL before injection. The separation was performed on a DB-5 capillary column (Agilent technologies, USA; 30 m × 0.25 mm ID, 0.25 µm film thickness). Helium was used as a carries gas at a constant flow rate 1.5 ml/min. The inlet temperature was 250°C, splitless injection mode. The oven temperature program was held at 40°C for 1 min and then raised to 210°C at a rate of 5°C/min, and finally to 250°C at a rate of 20°C/min. The mass spectrometer was operated in electron ionization mode at 70 eV, source temperature 230°C and quadrupole temperature 150°C.

### Compounds identification and semi-quantification

VOC identification was undertaken by comparing the recorded mass spectra using Wiley library. The peak identification was performed when the similarity of mass spectra was higher than 80%. The linear retention index was used to secure the identification of each molecule. Retention index was calculated by injecting saturated n-alkanes standards solution C7–C30 (Supelco, Belgium) using the definition of Kovats retention [49]. The identification was not confirmed using standards due to limited availability of chemicals. Absolute peak areas were used to calculate the percentage of each compound in the sample. The percentage was performed comparing the sum of peaks areas (hundred percent of compounds, including unidentified compounds) and the individual area of each compound.

### Histochemical localisation of terpenoids

For each O_3_ exposure time (three, five or seven days), three leaves from the sixth oldest node were used. The medial region of fresh, fully expanded leaf blades was freehand sectioned. Five sections were used for *in situ* localization of terpenoids using Nadi reagent (naphthol+dimethyl-paraphenylenediamine) [50]. Sections were incubated in the dark for 60 min at room temperature in Nadi reagent, prepared immediately prior to staining. After incubation, the sections were rinsed for 2 min in a sodium phosphate buffer (0.1 M, pH 7.2). By oxidation this reagent forms indophenol blue that changes colour with variation in pH and makes it possible to distinguish between essential oils (blue) and resin acids (intense red), in which a purple color is observed when both compounds constitute the secretion [50], [51]. Five other sections remained untreated – not submitted to any dye or reagents – for observation of the colour and structure of the cells *in vivo*. Observations and digital images were acquired with an Olympus BX53 compound microscope equipped with an Olympus Q-Color 5 digital camera and Image Pro Express 6.3 software.

### Identification and quantification of calcium oxalate crystals

The same leaves used for *in situ* localisation of terpenoids were used for COC quantification purposes. The remainder of each leaf was fixed in FAA_50_ (formalin-acetic acid-50% ethanol, 1∶1∶18) for 24 h and then stored in 70% ethanol [52]. The samples were hydrated and diaphanised using 10% sodium hydroxide and 20% hypochlorite solution (modified from) [52], and the medial region (approximately 3 cm in length) was sectioned and mounted in glycerine. Thirty squares of 5 mm^2^ were observed between crossed polarisers and photographed with an Olympus BX53 microscope. All the crystals present on images were counted using Image J (National Institutes of Health, USA).

For anatomical purposes, we used all the methods above on fully expanded leaves assuming that ozone symptoms initially appear with greater severity in older leaves toward the base of the plant [53] and that total terpenoids significantly increase with plant ontogeny [54].

### Is methyl salicylate responsible for the induction of COC?

To verify if methyl salicylate (MeSA) was directly involved in the induction of COC, we performed an independent experiment using young plants acquired from the same company, planted, watered, and two days acclimated in fumigation chambers as aforementioned.

Twelve plants were used for this experiment, separated in two groups of six plants and maintained separately inside two chambers, the same ones used for fumigation experiments, but emitting filtered air only. Plants were kept under the same conditions as described for filtered air plants from fumigation experiments during seven days.

A solution of 5 mM of MeSA (Sigma-Aldrich, Saint Louis, USA) was sprayed to each plant of the treated group every morning (1 ml/day), while distilled water was applied to each plant of the control group. After five and seven days of application the median region of the expanded leaves from two treated and two control plants were removed and then proceeded the protocols of COC counting as aforementioned. Thirty squares of 1.225 mm^2^ were observed between crossed polarisers, photographed and counted as described above.

### Statistical analysis

We used the statistical package SPSS 14.0 for Windows (SPSS; Chicago, IL, USA). Differences between FA and FA+O3 for antioxidant and physiological parameters were determined by the paired *t*-test with 95% confidence. VOC and COC were analysed using Repeated Measures ANOVA, with time and ozone as factors. Repeated Measures ANOVA was also applied on the independent experiment with MeSA and time. The differences between means were considered to be statistically significant at *P<*0.05. As the sphericity assumption was violated in all Repeated Measures ANOVA applied on the VOC data, the Greenhouse-Geisser conservative *F-test* was used [55].

The correlations among treatments, antioxidants, VOC emission and COC formation were investigated by principal component analysis using the data of seventh day of exposure in order to include all the variables in the statistical analyses (PCA, developed by PCord package 6.0), in which seven principal components explained over 90% of the variation.

## Results

### Which are the effects of ozone on antioxidant defence and fluorescence?

After seven days of exposure, there was a slight increase in total ascorbic acid and peroxidases in plants exposed to FA+O3 treatment, but no significant differences were found when these data were compared to FA treatment (Table 1). Significant decreases in the values of Fv/Fm and ETR (8.3% and 18.9%, respectively) and increase in the values of NPQ were observed in plants submitted to FA+O_3_ treatment.

**Table 1:** Values (mean and standard deviation) of biochemical and physiological parameters measured in leaves of *Croton floribundus* exposed to ozone (FA+O_3_) and control plants under filtered air (FA).

Variable	Treatment	
	FA+O_3_	FA
*Biochemical*		
AA (mg g^−1^ DW)	7.74±3.39a	7.70±1.85a
POD (10^2^ DA min^−1^ DW)	11.13±1.72a	10.55±0.80a
SOD (10^2^ U g^−1^ DW)	6.99±1.24a	7.90±0.89a
*Physiological*		
NPQ	0.657±0.021a	0.077±0.034b
Fv:Fm	0.654±0.017b	0.713±0.004a
ETR	82.7±8.8b	101.9±1.9a

Different letters indicate statistically significant differences among treatments for each parameter analysed (p<0.05).

### What are the emitted volatiles?

All the VOC identified were grouped into their respective chemical classes and summarised on Table 2. The relative quantity of oxygenated non-terpenoids and monoterpene tended to be higher in FA+O3 plants, representing 39 and 27% of total relative quantity of VOC emitted, respectively. MeSA, (E)-3-octen-2-one and 1,8 cineole showed higher levels in FA+O3 plants. No significant effects (*P*>0.05) of time and in the interaction between time and O_3_ were found.

**Table 2:** Percentages of volatile organic compounds (means ± S.D) emitted by leaves of *Croton floribundus* exposed to ozone (FA+O_3_) and control plants under filtered air (FA), and their linear retention indices in literature (Ri ref) and calculated (Ri cal).

Compounds	FA^a^	FA+O_3_ a	Ri ref	Ri cal^b^
*Non-terpenoid Oxygenated*				
nonan-2-one	0.67±0.48	0.53±0.52	1091	1091.3
pentadecanoic acid	0.33±0.15	0.50±0.35	1829	1826.3
Nonadecanal	0.33±0.21	0.37±0.24	2105	2105.1
octadecanoic acid	0.34±0.24	0.47±0.17	2200	2200.2
(E)-3-octen-2-one	2.20±1.70	4.07±1.21	1034	1033
*Sum*	*3.87*	*5.94*		
*Aromatic*				
(Z)-3-hexenyl benzoate	0.63±0.44	0.48±0.18	1570	1570,27
ethyl benzoate	0.42±0.22	0.62±0.22	1169	1170.68
methyl salicylate	0.30±0.32	0.73±0.13	1191	1191.13
methyl benzyl formate	0.57±0.28	0.57±0.26	1335	1335.56
methyl 3–4 dimethylbenzoate	0.52±0.41	0.34±0.23	1287	1353.73
2,6 dimethylphenol	Nd	0.25±0.20	1510	1504
*Sum*	*2.44*	*2.99*		
*Monoterpene*				
Ocimene	0.55±0.48	0.65±0.29	1063	1098
Geraniol	0.39±0.26	0.68±0.22	1276	1235
geranil acetate	0.32±0.12	0.54±0.25	1383	1378
γ terpinene	0.43±0.26	0.50±0.33	1016	1027
Carvone	0.42±0.30	0.45±0.14	1253	1248
Linalool	0.32±0.15	0.26±0.16	1173	1174
α-pinene	0.26±0.20	0.31±0.23	934	–-
1,8 cineol	0.70±0.37	1.47±0.18	1027	1030
Myrcene	0.34±0.19	0.40±0.25	988	––
*Sum*	*3.73*	*5.26*		
*Sesquiterpene*				
trans-nerolidol	0.53±0.40	0.55±0.18	1566	1564.2
β-gurjunene	0.48±0.37	0.56±0.26	1409	1423
caryophyllene oxide	0.70±0.26	0.84±0.25	1566	1576
aromadendrene	0.48±0.37	0.39±0.26	1636	1638
Copaene	0.55±0.26	0.58±0.29	1369	1372.8
α-caryophyllene	0.23±0.20	Nd	1411	1420
*Sum*	*2.97*	*2,92*		
**Significance of Repeated Measures ANOVA**				
**Factor** c	**F**		***P***	
ozone	0.02		0.90	
time	0.54		0.51	
ozone * time	0.94		0.49	

nd not detected.

a mean percentage and standard deviation of three replicates (including the six young plants after 7 days of exposure).

b linear retention index calculated on DB5 capillary column with a homologous series of n-alkanes (C8–C30).

--data insufficient to calculate Ri.

c Greenhouse-Geisser values calculated by ANOVA considering all volatiles.

### Where are the terpenes?

**Figure 1:**
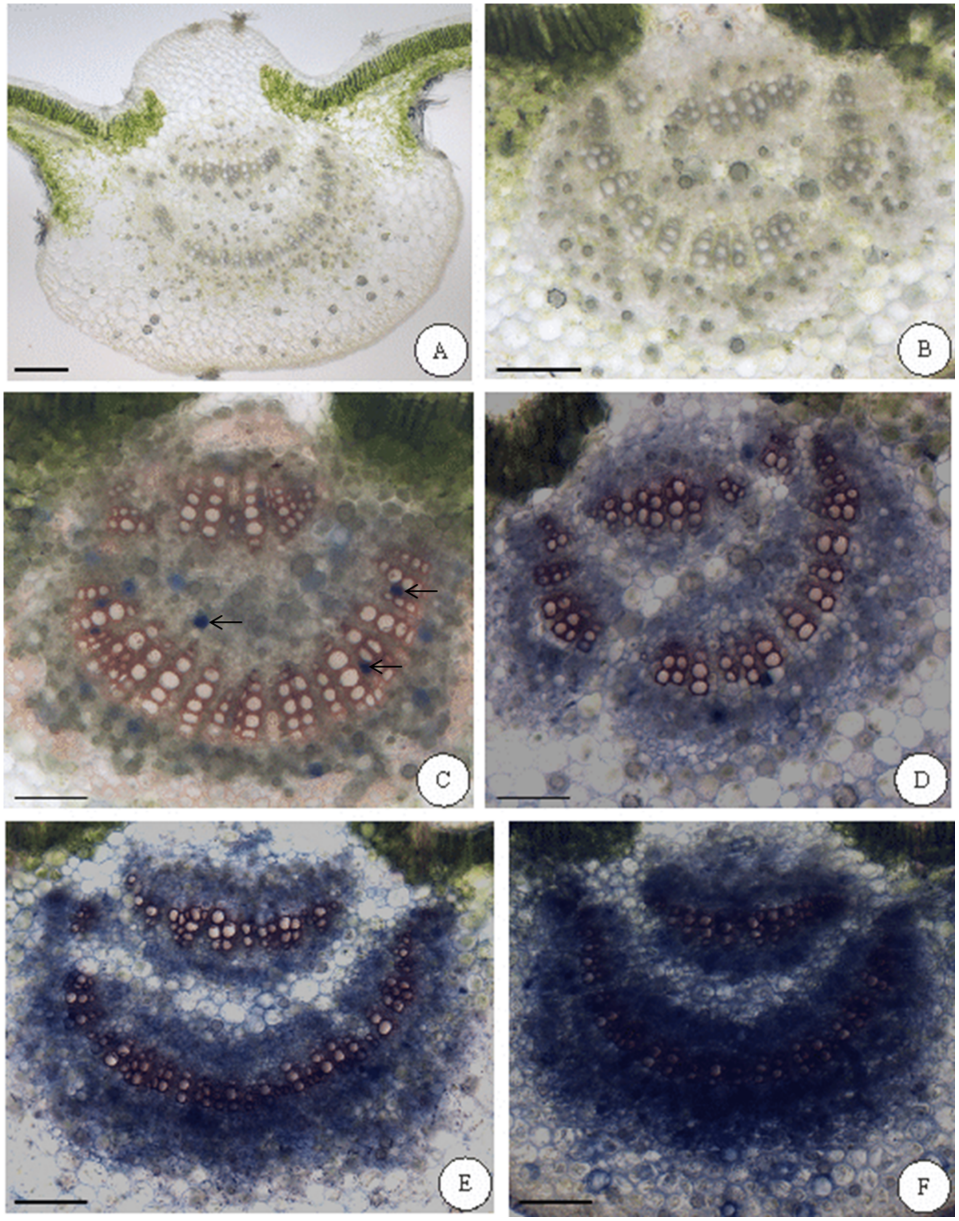
*In situ* localisation of terpenoids on leaves of *Croton floribundus*. Control plants under filtered air (A, C) and exposed to ozone for three (D), five (E) or seven (B, F) days. (A, B) Control from histochemical analysis (material without any dye or reagent). (C–F) Positive results for Nadi reagent inside laticifers (C); note a qualitative increase in the terpenoid content inside the parenchyma cells of vascular tissue according to the ozone exposure time (D–F). Arrows indicate laticifers. Bars: 100 µm.

Major qualitative results were observed in the midrib of the medial region of the leaf blade. Plants from FA and FA+O3 treatments presented no structural differences in epidermis, ground and vascular tissues (Figures 1A, B). The histochemical tests with Nadi reagent showed a positive reaction inside laticifers and parenchymal cells of xylem and phloem in all plants analysed: there were few cells with terpenoid content in leaves from FA treatment plants (constituent terpenoids, Figure 1C), while a qualitative increase of parenchyma cells involved on terpenoids metabolism was observed with increased exposure time to ozone (induced terpenoids, Figures 1D–F).

### Do ozone and MeSA increase the COC abundance?

**Figure 2:**
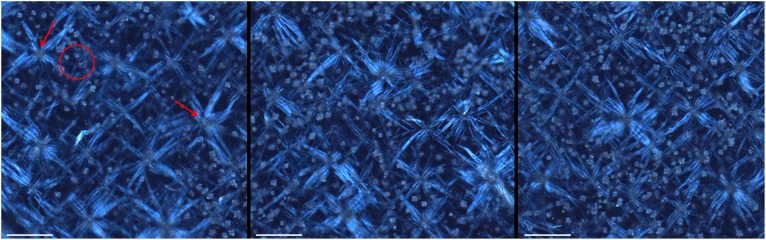
Areas in the adaxial surface of *Croton floribundus* observed under light microscope between crossed polarisers. Leaves before the exposition to O_3_ (A), and after five (A) and seven (C) days of exposition. Note the abundance of crystals (circle) and non-secretory stellar trichomes (arrows). Bars: 300 µm.

The abundance of COC and non-secretory stellar trichomes can be visualized in the Figure 2, also representing the areas where the quantification of the crystals was performed. Ozone fumigation resulted in a significant increase in the COC quantity (*P*<0.01) on the fifth and seventh days of exposure (Table 3). No significant effect of time (*P*>0.05) was observed on the COC quantity, whereas the effect of the interaction between O_3_ and time was significant (*P*>0.05).

**Table 3:** Effects of ozone, methyl salicylate and time (days) on the quantity of calcium oxalate crystals from *Croton floribundus* leaves.

Ozone treatment		
	Time (days) of exposure	
	5 (means ± SE)	7 (means ± SE)
FA	565±72	501±177
FA+O3	575±90	787±150
**Significance of Repeated Measures ANOVA**		
**Factor** a	**F**	***P***
ozone	31.42	0.001
time	1.77	0.192
ozone * time	27.12	0.001
**MeSA treatment**		
	**Time (days) of application**	
	**5 (means ± SE)**	**7 (means ± SE)**
Control	72.2±16.2	71.5±19.9
MeSA	105.6±14.7	111.53±21.9
**Significance of Repeated Measures ANOVA**		
**Factor** a	**F**	***P***
MeSA	165.67	0.001
Time	0.85	3.463
MeSA * time	1.37	0.243

a Greenhouse-Geisser values calculated by ANOVA.

A significant increase was observed in the COC quantity on leaves from plants treated with MeSA (*P*<0.01; Table 3): the increase was not time-dependent and there was no interaction effect between time and MeSA.

### Correlations among antioxidant defence, volatiles and crystal formation

**Figure 3:**
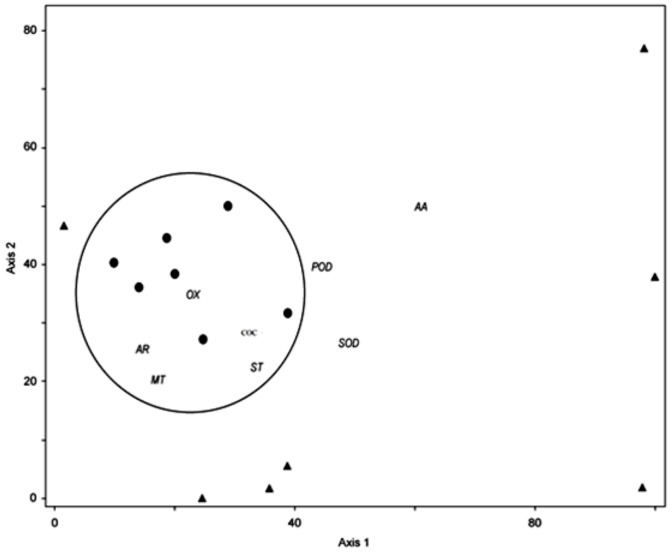
The PCA biplot diagram showing the volatile organic compounds (VOC) and antioxidant defences superimposed on the score of treatments indicated by black circles (ozone exposed, FA +O_3_) and black triangles (filtered air, FA). The PC1 axis separates the treatments based on the O_3_-induced VOC and calcium oxalate crystals (COC). On the PC2 axis, the treatments were characterised by their antioxidant defences. Abbreviations: VOC as aromatic (AR), monoterpene (MT), oxygenated (OX), sesquiterpene (ST); Ascorbic acid (AA); Peroxidases (POD); Superoxide dismutase (SOD).

Principal components analysis was used to study relationships among the antioxidants, volatiles and crystal formation (Figure 3). The first PC was described by the term ‘antioxidant defence’ and explained 57% of the total variation in the data, characterised by a high score of ascorbic acid rather than SOD and POD. The second PC, designated as ‘induced defence’, explained 26% of the total variation and was characterised by a lower score in volatile and crystal levels than ascorbic acid. The treatments were separated into specific groups: FA treatment was more related to antioxidant defences, explained by a high level of AA, while FA+O3 treatment was linked to volatile emissions and crystal formation.

## Discussion

Our results indicate that O_3_ induces biochemical, physiological and anatomical responses in a native Brazilian species. When these responses are combined, it is possible to understand how the plant can defend itself and how apparently independent features, such as the emission of VOC and production of COC, may play an important role in these responses.

The antioxidant defences act against O_3_ to avoid the increase in cellular ROS such that the expression and/or activity levels of enzymes and other compounds change to keep the pro-antioxidant balance [13], [56]–[61]. Changes in the antioxidant levels, such as an increase in the level of ascorbate as a response to O_3_ fumigation, have been reported in spinach (*Spinacia oleracea*) and beech (*Fagus sylvatica*) [59]. In contrast, when the O_3_-induced ROS does not result in a reducing environment inside the cell, the antioxidant levels might remain constant. In this case, there can be increases in other metabolic compounds that can act as scavengers of ozone before its strong oxidative action occurs in the cell [1], [16], [62].

In our study, no significant changes were observed in the levels of antioxidants whereas the inducible defences, terpenoid compounds, tended to increase in O_3_ exposed leaves. This can be explained by the fact that O_3_ uptake does not necessarily cause injury in plants due to the production of metabolic compounds, such as volatiles, that remove ROS before they cause serious damage in the cell [63].

In our experiment, although there was a slight increase in the quantity of volatiles emitted, there was no change in volatile profile and *de novo* volatiles were not induced. Monoterpenes, aromatics and non-terpenoid oxygenated compounds, which have been reported to be protective against oxidative damage due to rapid reaction with ROS [15], [18]–[20] increased with FA+O3 treatment. Thus, the increase in these constitutive metabolites in *C. floribundus* might be a defence response against O_3_ damage, removing ROS through gas-phase chemical reactions in the intercellular spaces of the leaves [2] because isoprene synthesis is stimulated by ozone [11], [64]. Although terpenoid compound levels might have inhibited the effects of ozone most likely by restricting the damage caused by ROS, the fumigation of *C. floribundus* with 80 ppb of O_3_ lead to a significant decrease in Fv:Fm and ETR compared to the FA condition, which might indicate a reduction of photosystem II (PSII) efficiency. A decrease in the ability of PSII to dissipate light energy as non-photochemical quenching (NPQ) can induce the ROS production in chloroplast, signalling the primer mechanism of immune response [65]. In addition, chloroplast-generated ROS are important to up-regulation of defence-relate genes and down-regulation of photosynthesis, such as the regulation of redox state of photosynthetic components, including plastoquinone and glutathione pools [65], [66]. Our results suggest no disturbance in enzymes and ascorbic acid, which are compounds of ascorbate-glutathione cycle, indicating that ROS produced by O_3_ likely not disturbed the redox state of the chloroplast. Our results also indicate that the ROS produced by ozone exposure in *C. floribundus* might have been sufficient to signal the terpenoid pathway but not to cause a strong oxidative stress.

Secretory glands such as laticifers are involved in plant defences against herbivory [67], [68]. Laticifers’ metabolite contents are biochemical end products (generally cytotoxic); these cells are involved in sequestering toxic compounds or their precursors independent of vascular tissues [69]. It is assumed that laticifers have no functional plasmodesmatal connections with their neighbour cells [70]–[71], making it impossible to release their contents without physical injury, and they are not modulated in response to oxidative stress. On the other hand, terpenoids are produced by cells of the mesophyll and parenchyma cells from the vascular system, which are also responsible for translocation of these compounds [28]–[30]. Indeed, FA treatments presented only laticifer cells with terpenoid content (constituent terpenoids), but a progressive recruitment of vascular parenchyma cells involved with terpenoid production and translocation was observed in the course of the time in which the plants were exposed to O_3_. Since the terpenoids are volatile, their diffusion from these cells can be confined over and to the leaf (boundary layer) by means of the non-secretory trichomes, which are abundant in both faces of the epidermis. These stellar non-secretory trichomes can act as a container for volatiles analogous to the corona on flowers of *Passiflora* species [72]. Therefore, the concentration of VOC under the leaf might be increased simply by the presence of these non-secretory trichomes. Because VOC can scavenge O_3_, this morphological adaptation can also prevent the entrance of this pollutant into the leaf.

Considering that the production of volatiles is systemic [73]–[74], the O_3_-induced VOC in *C. floribundus* might be transported by phloem throughout the plant and act as signalling defences. Methyl salicylate, an oxygenated volatile that increased when *C. floribundus* plants were exposed to O_3_, is one of the key messenger molecules synthesised by plants in response to stress [75]. This compound may act as a mobile signal throughout the plant, triggering the systemic acquired resistance by means of its precursor, salicylic acid, which is able to enhance chemical defences, such as antioxidants [75]–[76]. It is interesting to note that ROS is mediated by salicylic acid, which plays a key role in the changes in the cytosolic concentration of free calcium ions [77]. Indeed, O_3_ stress induces the ROS-mediated opening of calcium channels and increases the intracellular calcium concentration [78], a mechanism involved in COC formation [79]. In our work, *C. floribundus* exposed to O_3_ showed slightly increase in MeSA emission and in the number of COC, but there is no evidence of alteration in pro-antioxidant balances. Therefore, we hypothesized that the ozone-induced VOC (such as MeSA) regulate ROS formation in a way that promotes the opening of calcium channels and subsequently, the formation of crystals. To test this hypothesis we performed an independent experiment spraying MeSA on *C. floribundus* leaves and verified that, indeed, MeSA was able to induce an increase on COC number. Thus, we suggest that the increase in the quantity of crystals could be a fast and stable mechanism to stop the consequences of ROS in the tissue, immobilising the calcium excess that can be dangerous to the plant.

Our findings indicate that the tolerance of *C. floribundus* to oxidative stress caused by ozone may be the consequence of a higher capacity to produce volatiles and oxalate crystals rather than the modulation of the antioxidant balance. These metabolic features could be used as biomarkers for ozone tolerance and may also be useful for choosing the functional groups resistant to air pollution and, consequently, for use in ecological restoration plans in urban areas highly impacted by air pollution.
